# Vicarious ratings of social touch the effect of age and autistic traits

**DOI:** 10.1038/s41598-021-98802-2

**Published:** 2021-09-29

**Authors:** Connor J. Haggarty, David J. Moore, Paula D. Trotter, Rachel Hagan, Francis P. McGlone, Susannah C. Walker

**Affiliations:** 1 Research Centre for Brain and Behaviour, Liverpool John Moores University, Liverpool, UK; 2 Centre for Social and Affective Neuroscience, Linköping University, Linköping, Sweden; 3 Institute of Psychology, Health and Society, University of Liverpool, Liverpool, UK

**Keywords:** Psychology, Human behaviour

## Abstract

Tactile sensitivities are common in Autism Spectrum Conditions (autism). Psychophysically, slow, gentle stroking touch is typically rated as more pleasant than faster or slower touch. Vicarious ratings of social touch results in a similar pattern of velocity dependent hedonic ratings as directly felt touch. Here we investigated whether adults and children’s vicarious ratings vary according to autism diagnosis and self-reported autistic traits. Adults’ scoring high on the AQ rated stroking touch on the palm as less pleasant than a Low AQ group. However, in contrast to our hypothesis, we did not find any effect of autism diagnosis on children’s touch ratings despite parental reports highlighting significant somatosensory sensitivities. These results are discussed in terms of underpinning sensory and cognitive factors.

## Introduction

Differences in sensory processing is a core diagnostic feature of autism^1^. Clinical estimates of the prevalence of sensory differences in children and adults range from 30 to 100%^2,3^, with a large portion being specifically related to tactile sensitivity^4,5^ Indeed, parental and clinician reports frequently highlight hyper and hypo-responsivity to touch in autistic children and the strength of the sensory sensitivity is predictive of later life levels of social function^6^. However, how differences in sensory perception relate to the social communicative and cognitive difficulties which characterize the condition is not yet understood^7,8^. To date, research on sensory perception in autism has focused primarily on the visual and auditory domains, with very little consideration of the impact that tactile sensitivities play in the development and wellbeing of individuals. Touch is the first sensory system to develop in utero and the key sensory channel through which early social interactions occur, so different processing of somatosensory information could have a significant impact on the developing social brain^9^.

Processing of somatosensory stimuli has both a discriminative and affective dimension^10,11^. In pain this distinction is termed ‘first’ and ‘second’ pain. Perceptually, first pain is experienced as a brief sharp, prickling burning sensation, signalled by fast conducting myelinated Aδ/Aβ afferents which elicit a rapid reflexive withdrawal from danger^12^. In contrast, second pain is a longer lasting, dull, burning sensation which motivates protective behavior promoting healing^13^. It is only more recently that a similar discriminative/affective distinction has been proposed for the sense of touch^14,15^. Discriminative aspects of touch are signalled by myelinated Aβ fibers which facilitate rapid detection and localization of touch on the body and is required for haptic exploration and manipulation of objects. In comparison, affective aspects of touch are signalled by unmyelinated low threshold mechanoreceptors. These afferent fibers conduct too slowly to provide useful discriminative information and, in common with other C-fibers, they are hypothesized to perform a protective, homeostatic and pro-social function^16,17^.

The combination of electrophysiological and psychophysical methods has provided interesting insights into the unique response characteristics of the C-low threshold mechanoreceptors which innervate the hairy skin of humans (i.e., all locations except those with glabrous skin such as the palm of the hand and soles of the feet). Named C-Tactile afferents (CTs), these nerve fibers are velocity and temperature tuned, responding most strongly to a skin temperature stimulus moving across their receptive field at between 1 and 10 cm/s. In psychophysical studies, typically developing participants’ hedonic ratings of gentle moving touch are correlated with CT firing frequency^14,18^. That is, on a group level, the highest pleasantness ratings are reliably given to a stimulus moving across the skin at 1–10 cm/s^14,18^. These response characteristics, coupled with central projections to limbic brain regions, led to the proposal of the ‘social touch hypothesis’, that CTs evolved to signal the rewarding value of social tactile interactions and thus promote protective affiliative behaviors^19,20^. Support for this hypothesis comes from the observation that parents spontaneously caress their infant at a CT optimal velocity^21–23^.

The neural projections of this CT optimal touch have been shown to be to regions such as anterior insula, orbitofrontal cortex and the medial prefrontal cortex which are also involved in key aspects of social perception and cognition^24–26^. It is also noteworthy that the functioning of these regions and their underlying social processes are different in autism^27,28^. Attesting to the relationship between processing of socially relevant tactile stimulation and social-cognitive functioning, the frequency of maternal touch has been found to predict resting state activity and connectivity in key nodes of ‘the social brain’. In five-year-old children a positive correlation was found between the amount of tactile interaction they received from their mother during 10-minutes of play and subsequent resting activity and connectivity in a network of regions including, the posterior Superior Temporal Sulcus (pSTS), Temporal Parietal Junction (TPJ) and Insula^29^. In adults, individual differences in this network’s response to CT-targeted touch are correlated with psychophysical ratings of CT targeted touch^21,30–33^.

Given this potential link between tactile processing and social cognition, several studies have investigated whether processing of affective aspects of touch are different in autistic children and adults^34^. Neural differences in processing of affective touch have been reported in adults with autism, reflected as blunted neural responses in affective nodes of the social brain^32,35^. In neurotypical populations the magnitude of response correlates negatively with self-report measures of autistic traits^33,36^. However, in psychophysical tests the evidence for different in responding is more mixed, with several studies reporting increased interindividual variation but otherwise typical ratings of affective tactile stimulation in autistic adults^35,37^. Though, in a large cohort of neurotypical adults a negative correlation between self-reported autistic traits and sensitivity to the specific rewarding value of CT-optimal stroking touch has been reported^21^. Consistently, in autistic children, defensiveness towards socially relevant tactile stimulation were positively correlated with the children’s levels of social impairment^38^.

Empathy is a function of social behavior that allows an individual to understand and respond appropriately to other people’s cognitions and emotions^39^. Empathic responses to vicarious somatosensory experience have been widely studied with respect to pain [e.g.^40–42^]. For example, observation of a romantic partner in pain resulted in similar activation in the ‘pain matrix’ as seen when participants experienced the pain first-hand^42^. Vicarious responses can also be behavioral, for example, Lamm et al.,^43^ reported increased muscle activity, indicative of negative affect, when participants were asked to imagine themselves in the place of a patient they watched undergoing a painful procedure. Empathic responses to vicarious social stimuli may be different in individuals diagnosed with autism whereby understanding of other’s intentions and thoughts are often mistaken^44–46^. In the context of social touch, it is however currently unclear how empathic response to interpersonal touch may operate in autistic individuals with some evidence of preserved empathy for pain in others^44^, and recent hypotheses surrounding not an absence of empathy in autism but rather a ‘double empathy problem’^47^ hereby there is a mismatch in empathic responses between neurotypes, with autistic and NT neurotypes experiencing challenges in emotional decoding between the groups.

Mirrored neuronal responses have also been reported during observation of other’s non-painful somatosensory experiences^47,48^, such as socially relevant interpersonal touch^47^, including specifically CT targeted touch^19^. Furthermore, psychophysical ratings of observed touch have been reported to show the same relationship between stimulus velocity and perceived pleasantness as feeling that touch first-hand^49–51^. The fact that patients suffering from a rare congenital C-fiber deafferentation rate both directly felt and vicariously experienced CT-optimal touch as less pleasant than control participants^19^ indicates vicarious ratings are strongly influenced by personal affective experience of CT stimulation.

Developmentally, several recent neuroimaging studies have provided evidence that the CT system is active in early infancy^52,53^. Psychophysically children, like adults, show a preference for CT targeted over non-CT targeted touch both when it is directly experienced^54^ and vicariously viewed^55^. Therefore, the aim of the present study was to determine whether, in line with previous findings with directly felt touch, adults with high levels of autistic traits and children with a diagnosis of autism show blunted ratings of the hedonic value of vicariously experienced CT targeted touch. It is hypothesized that children and adults will similarly show a preference for CT-optimal stimuli over non-optimal stimuli and that both children and adults with high autistic traits or a diagnosis of autism will show blunted preferences for CT-optimal social touch compared to individuals with low levels of autistic traits and typically developing children.

- Experiment 1: Do adults with high versus low levels of autistic traits differ in their affective ratings of vicariously experienced social touch?

## Methods

### Participants

Ninety-six male participants aged 18–30 (Mean = 21.26, S.D. ± 2.49), were recruited via mailing lists at Liverpool John Moores University. To recruit a sample with a broad a range of AQ scores, emails were sent out to subject lists relating to science, technology, performing arts and English^56^. All participants who completed the experiment were entered into a prize draw to win a £50 gift voucher. This experiment received ethical approval from Liverpool John Moores University Research Ethics Committee and the experiment was conducted in accordance with the Declaration of Helsinki. Informed consent was obtained prior to participants completing the study.

### Measures

#### *Autism spectrum quotient* (*AQ*)^56^

The AQ measures autistic traits in the general population. The questionnaire consists of 50 statements and asks participants to indicate how much each one applies to them on a 4-point scale with descriptors: ‘Definitely agree’, ‘Slightly Agree’, ‘Slightly Disagree’ and ‘Definitely Disagree’. For half the questions an ‘Agree’ or ‘Slightly Agree’ response indicates characteristics similar to those on the autistic spectrum and are scored as 1, whereas ‘Disagree’ or ‘Slightly Disagree’ responses are scored as 0. The other 50% of questions are reverse scored.

#### Touch videos

**Figure 1:**
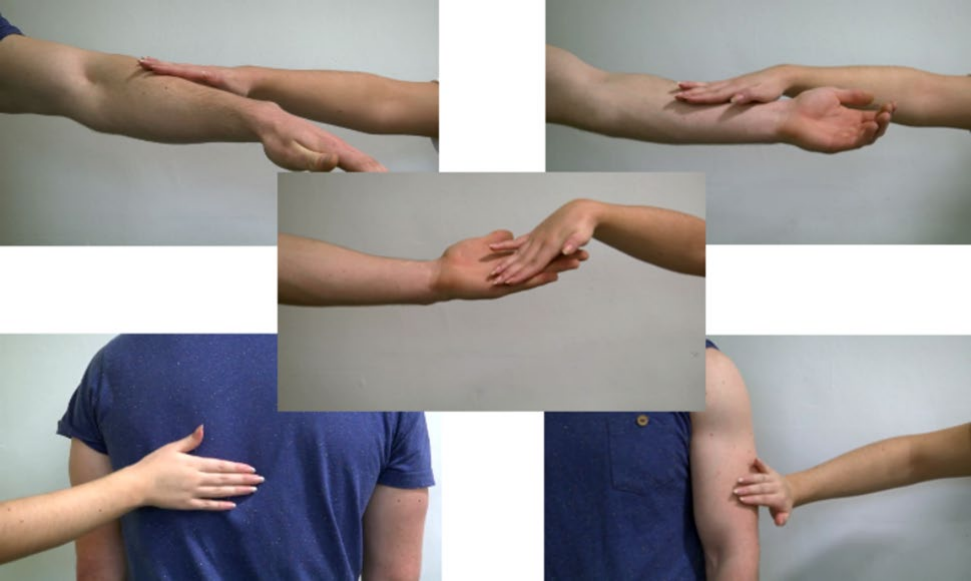
Stills from the videos presented, one depicting each of the 5 locations studied. The clips lacked any social context, faces were not visible, and showed only the hand and forearm of one female actor ‘the toucher’ and the relevant upper body part (back, arm or palm) or the other male actor ‘the receiver’.

Participants viewed and rated a sequence of 15 short (5 s) videos^51^ presented in a random order depicting one adult male actor being touched by an adult female actor at 5 different skin sites (back, upper arm, ventral forearm, dorsal forearm and palm) and at 3 different velocities (Static, Slow ~ 3 cm/sec, Fast ~ 30 cm/sec). (Fig. 1 shows video stills, depicting the 5 body sites investigated). Immediately after viewing each clip a new screen appeared where participants were asked to rate, on a Likert scale: (1) How pleasant do you think that action was for the person being touched? (2) How much would you like to be touched like that? Rated from 1 not at all—7 extremely. These two questions always appeared in the same order, each on a new screen, with question 2 appearing directly after the response to question 1 was made. They were designed to probe expectations of how touch is perceived by others versus self.

### Procedure

The Experiment was conducted online using Qualtrics software, Version 60,939 of the Qualtrics Research Suite. (Copyright © 2015 Qualtrics., Provo, UT, USA. http://www.qualtrics.com). Start and end time of survey completion was recorded. Mean time online was 11.7 min (S.D. ± 3.19 min). Participants were first presented with a screening questionnaire, asking them to confirm their age and gender. If they fulfilled the experiment’s inclusion criteria (male and over 18), they were then presented with a series of demographic questions relating to their ethnicity and current or previous mental health conditions (just three participants reported autism, these all appeared in the High AQ group). They then completed the AQ before watching and rating the videos.

### Data Analysis

**Figure 2:**
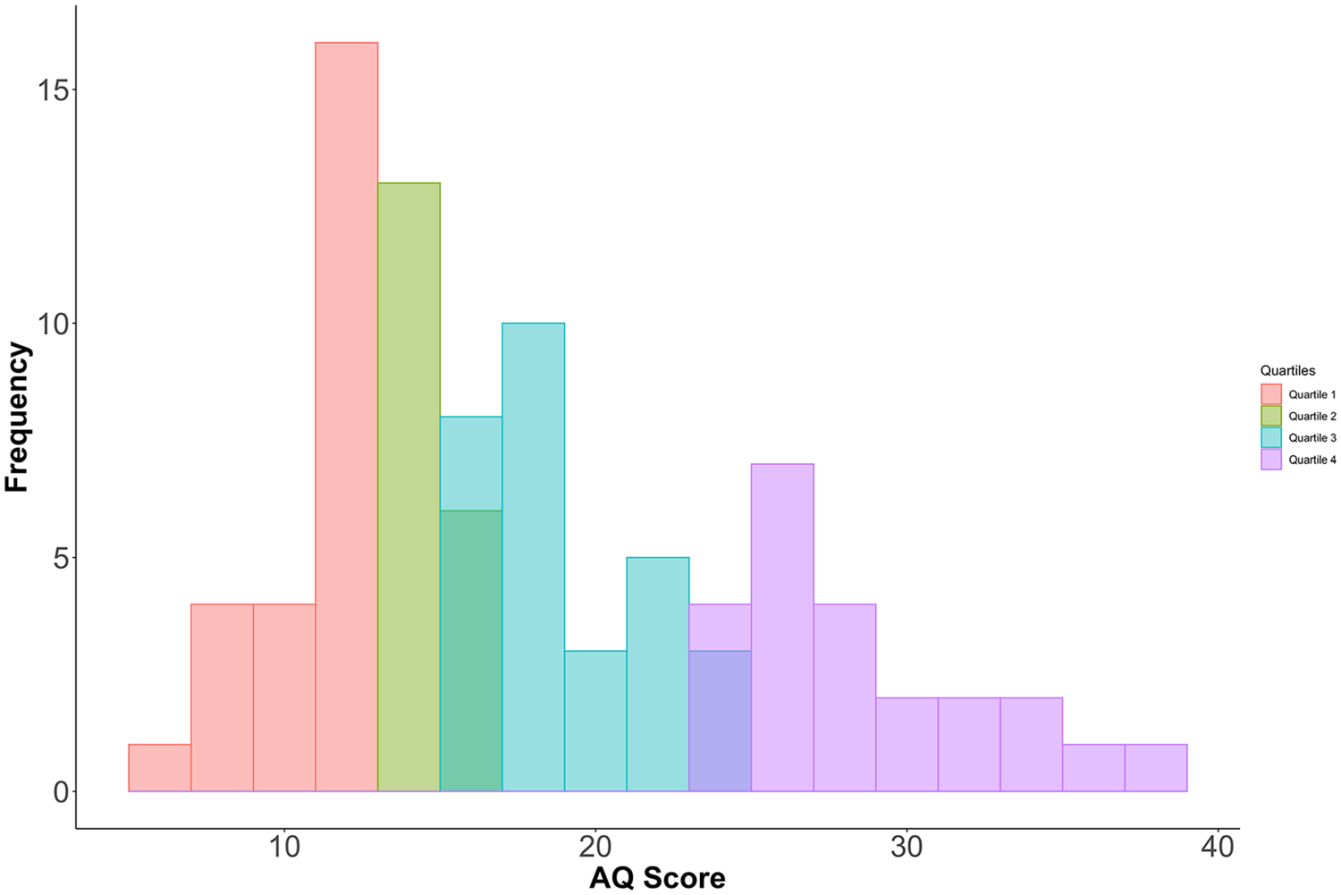
Frequency of AQ scores in the sample. (n = 96). Within the quartiles of interest, Individuals in the Low AQ group fall below a typical population average (N = 25, Mean AQ = 11.36, SD = 1.93 Range 5–13 in Quartile 1 (~ 17, Baron-cohen et al., 2001). Individuals in the High AQ group scored 26 or above are in Quartile 4 (N = 19, Mean = 30.05, SD = 3.96, Range 26–39). Previous studies have reported 80% of individuals diagnosed with autism also score 26 or above^59^.

Data were analyzed using SPSS (IBM Corp. Released 2013. IBM SPSS Statistics for Windows, Version 22.0. Armonk, NY: IBM Corp). Inspection of model residuals indicated data were normally distributed. Assumptions of sphericity were not violated. To correct for multiple comparisons, Bonferroni correction was applied. Since here it is assumed participants are rating pleasantness as a continuous variable and our data met the assumptions for parametric analyses^57,58^ initially data were analyzed using a repeated measures ANOVA with within subject factors of Location (5 levels) and Velocity (3 levels). Since, consistent with previous findings^51^, responses to the two questions (Vicarious Pleasantness & Desire) were found to be highly correlated, all *r* > *0.5* and p < 0.001, data were averaged across questions to produce a single dependent variable, affective touch ratings. Next, participants were separated into two extreme groups, according to their score on the AQ (Fig. 2) using a quartile split, so comparisons could be made between individuals with the fewest and the most autistic traits^60^. The median score of the sample was 17. All participants scoring 13 or under made up the Low AQ group (N = 25, Mean AQ = 11.36, SD = 1.93 Range 5–13), while all participants scoring 26 or over made up the High AQ group (N = 19, Mean = 30.05, SD = 3.96, Range 26–39), where a score of 26 is often noted as the lowest threshold for individuals diagnosed with autism^59^. Three participants in the High AQ group also had a diagnosis of autism however, there were typically developing participants in this group scoring higher on the AQ so these participants were included together. For the between group analysis, to minimize the number of variables, and thus increase power to detect effects of interest, a 2 × 2x2 mixed ANOVA was conducted. The factors were Group (High AQ v Low AQ), Location (Glabrous v Hairy Skin Locations) and Velocity (CT Optimal v Non-CT Optimal). Figures were drawn using R packages tidyverse^59^ and ggsignif^61^.

## Results

### Location by Velocity ANOVA

**Figure 3:**
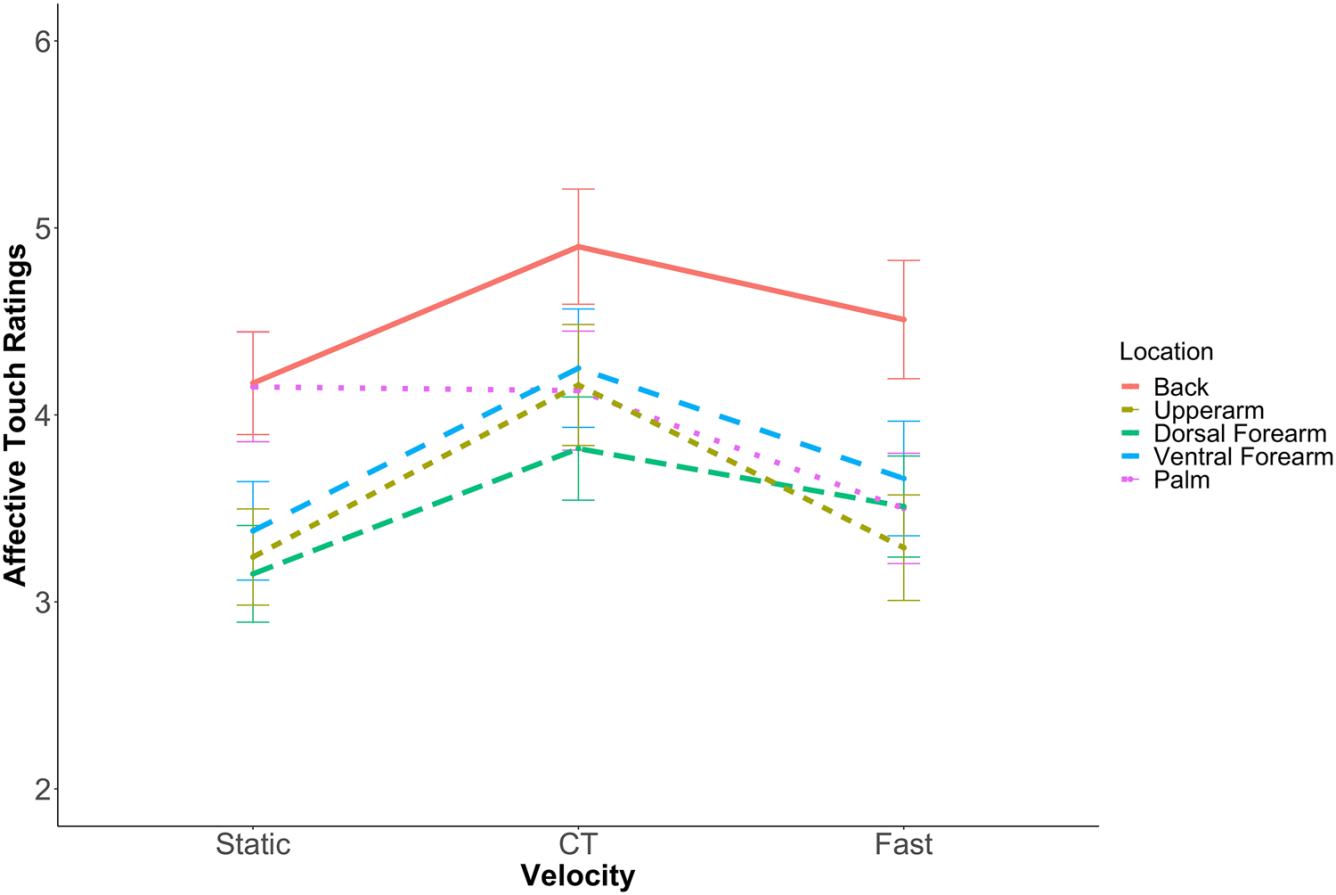
Data show the established quadratic relationship between affective ratings and velocity of touch on the dorsal and ventral forearm, upper arm and back. Here slow, CT-optimal (~ 3 cm/s) touch was rated more positively than static and fast (~ 30 cm/s) touch. In contrast, on the palm, static touch is rated equally positively to slow, CT-optimal (~ 3 cm/s) touch (bars ± 95% confidence).

A repeated measures ANOVA revealed a significant main effect of Location F(4,372) = 29.512, *p* < *0.0*01, η_p_2 = 0.24 and Velocity F(2,186) = 27.47, *p* < *0.0*01, η_p_2 = 0.23. These reflect significantly higher ratings for touch on the back and at the CT-optimal slow velocity (~ 3 cm/s) respectively. However, there was also a significant Location × Velocity interaction F(8,744) = 7.75, *p* < *0.0*01, η_p_2 = 0.07 (Fig. 3). To further investigate the interaction effect, ANOVAs were run to determine the relationship between Velocity and affective ratings at each skin site separately. At all Locations hypothesised to be densely innervated with CTs, (back and arms), slow, CT-optimal stimulation (~ 3 cm/s), was rated significantly more positively than static or fast touch (all *ps* < *0.0*01). Ratings of static and fast touch did not differ significantly from each other (all ps > 0.05). In contrast, on the glabrous skin of the palm, there was a different relationship between Velocity and affective ratings. While ratings of static and slow (CT-optimal, ~ 3 cm/s) touch did not differ significantly (*p* > *0.0*5), both were rated significantly more positively than fast (~ 30 cm/s) touch (ps < 0.001). These findings replicate the result of our previous experiment using these video stimuli^51^. Furthermore, Croy et al.^21^ reported a relationship between autistic traits and CT-preference, this was calculated for each Location using the equation (*CT-optimal* (3 cm/s) *– non-CT-optimal* (30 cm/s) * Σ(*Static, CT-optimal, nonCT-optimal*)*/*3). Therefore, here we calculated these CT-preference indices. However, we found that for the vicarious experience of touch no preference index correlated significantly with autistic trait scores (*rs − 0.1, ps* > *0.0*5).

Thus, in the following between group analysis ratings of touch at body sites where CT optimal velocity touch was rated as most pleasant (Back, Upper-arm, Dorsal and Ventral Forearm) were averaged together and considered in comparison to ratings of touch on the palm.

### Group (High/Low AQ) x Location (Hairy/Glabrous skin) x Velocity (Static/CT-optimal/non-CT-optimal)

A repeated measures ANOVA revealed a significant main effect of Velocity F(2,92) = 9.23, *p* < *0.0*01*,* η_p_^2^ = 0.17, reflecting the fact CT-optimal velocity stroking was rated most pleasant. However, there was no significant main effect of Location F(1,46) = 0.34, *p* > *0.0*5*,* η_p_2 = 0.02. While there was a significant main effect of AQ group F(1,46) = 8.24, *p* < *0.0*1 *,* η_p_^2^ = 0.15, reflecting that, on average, the Low AQ group rated touch as more positively than the high AQ group, there was also a significant Location by Velocity by AQ group interaction F(2,92) = 5.37, *p* < *0.0*1 *,* η_p_^2^ = 0.11.

**Figure 4:**
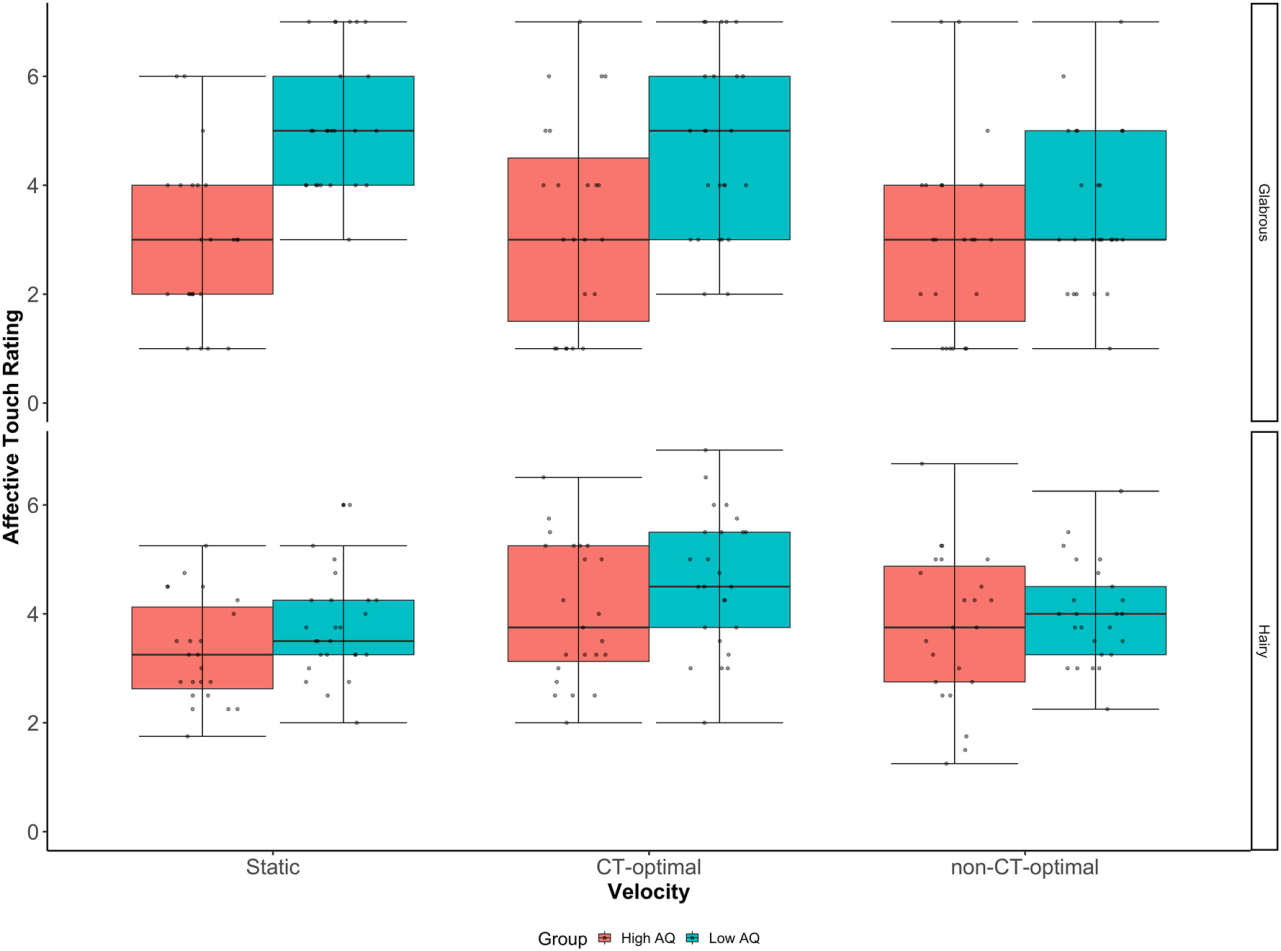
Box plot showing the difference between groups for CT vs nonCT velocities in the ratings of touch on the hairy (Back and Arm) and glabrous (palm) skin locations. Participants with the highest number of autistic traits rate touch to the Palm significantly lower than individuals with the lowest number of autistic traits for both Static and CT-optimal velocities (bars ± 95% confidence).

To further investigate the interaction, Velocity × Group ANOVAs were run separately for the hairy skin sites (arm and back) and the glabrous (palm) skin site. For hairy skin sites, there was a significant main effect of Velocity F(2,92) = 20.90, *p* < *0.0*01 *,* η_p_^2^ = 0.31 driven by higher ratings for CT-optimal velocity touch (M = 4.32, SD = 1.30) compared to Static (M = 3.52, SD = 0.94, *p* < *0.0*01) and non-CT-optimal velocity touch (M = 3.88, SD = 1.16, *p* < *0.0*01). However, there was no significant main effect of group (*p* > *0.0*5). Comparatively for the palm there was a significant main effect of both Velocity F(2,92) = 7.75, *p* < *0.0*1 *,* η_p_^2^ = 0.14 and Group F(1,46) = 13.30, *p* < *0.0*1*,* η_p_^2^ = 0.22 as well as a Velocity by Group interaction F(2,92) = 6.66, *p* < *0.0*1*,* η_p_^2^ = 0.13. Simple main effects analyses for each Velocity, revealed a significant difference between High and Low AQ groups for both static (*p* < *0.0*01) and CT-optimal (*p* < *0.0*1) touch where individuals in the Low AQ group rated these stimuli significantly more positively than individuals in the High AQ group. However, there was no difference between the groups in their ratings of the fast, non-CT-optimal velocity touch (*p* > *0.0*5 (Fig. 4).

## Discussion

In the first experiment, touch observed at CT-optimal velocity on hairy skin upper body sites was rated as more pleasant than non-CT-optimal, slower and faster, velocity touch. This was not the case with ratings of touch on the palm, where static and slow, CT-optimal touch were rated as equally pleasant. Furthermore, touch on the back was rated as more pleasant than touch on the arm or palm. These findings are consistent with the findings from our previous studies using these video stimuli^50,51^ and are hypothesized to reflect variation in of C-fiber density across these skin sites^62,63^.

Both the high and low AQ groups showed a preference for CT-optimal velocity touch over non-CT-optimal touch on hairy skin sites. Though overall the high AQ group’s hedonic ratings were lower than the Low AQ group, this was primarily driven by significantly lower ratings of touch viewed on the glabrous skin of the palm. Here, the high AQ group rated CT-optimal and static touch as less pleasant than the low AQ group, but not non-CT-optimal fast touch. These findings suggest that differences in ratings of touch in those with high levels of autistic traits are body site rather than velocity specific. Though this is inconsistent with previous neuroimaging studies which have reported neural responses to different velocities of touch on the hairy skin of the arm vary according to levels of autistic traits^33,36,64^, they are consistent with previous reports in psychophysical studies that affective touch ratings don’t differ between those with and without an autism diagnosis [e.g.^37,65^] or those with high and low autistic traits^33^, though see Croy et al^21^.

It has been hypothesized that this discrepancy between psychophysical ratings and neural responses to directly felt affective touch may be the result of experience, with adults displaying typical behavioral responses despite varying neural processing^38^. Research to date indicates neurotypical children’s neural and behavioral responses to affective touch are similar to adults^65–68^. Thus, in Experiment 2 we recruited children with and without a clinical diagnosis of autism to compare their affective ratings of vicariously experienced social touch. We hypothesized children with a diagnosis of autism would rate touch as less pleasant and would be less sensitive to the specific rewarding value of CT optimal velocity touch than their neurotypical peers.

- Experiment 2: Do autistic children differ in their ratings of vicariously experienced social touch to neurotypical peers matched for age and verbal ability?

## Materials and Methods

### Participants

Twenty-seven children aged 7–12 years took part in the experiment. Thirteen (11 Male, Mean Age 9.3, S.D. ± 1.70), were recruited through a community organization in the North West of England and had received a diagnosis of autism by a trained clinician based on DSM^69^ criteria. Parents/Guardians gave written informed consent for their child’s participation. Each child also provided informed assent before beginning the experiment. In addition, fourteen typically developing children (8 Female, Mean Age=8.3, S.D. ±1.2) were recruited from a year four class (typical age 8–9) at a primary school in the North West of England. Written informed consent was given by parents prior to the students completing the experiment. Each child also provided informed assent at the beginning the experiment. This experiment received ethical approval from Liverpool John Moores University Research Ethics Committee and the experiment was conducted in accordance with the Declaration of Helsinki. All children received a LEGO® toy as a thank you taking part.

### Materials

#### *The British picture vocabulary scale—second edition* (*BPVS-II*)

Was used to ensure that there were no significant group differences in receptive vocabulary. The BPVS-II is an untimed test of a child’s receptive vocabulary level for Standard English. On each trial the examiner reads a word, and the child is asked to select which of 4 pictures best illustrates the word’s meaning. Participants are first introduced to the test and then, based on their age, their basal set of stimuli (one on which they make one or no errors) is established. The test continues with word sets of increasing difficulty until a ceiling set (a set of stimuli on which they make eight or more errors) is reached^70^. A total of 14 sets of 12 items is available. Analyses showed no significant difference in BPVS scores between autism (M = 93.08, SD = 13.44) and control (M = 87.93, SD = 12.46) groups, t(25) =  − 1.03, *p* > *0.0*5*.*

#### Sensory profile

Parents of children in the autistic group were given the Sensory Profile (Dunn, 1999) to complete. The scale consists of 60 questions asking how the child responds to sensory experiences at home. Individual questions refer to a single sensory modality. For these data, the SP subscale for touch sensation was extracted. For example, “reacts emotionally or aggressively to touch” and “touches people and objects”. Touch sensation scores ranged from 40–75 (M = 54.23, SD = 12.64).

#### Touch videos

**Figure 5:**
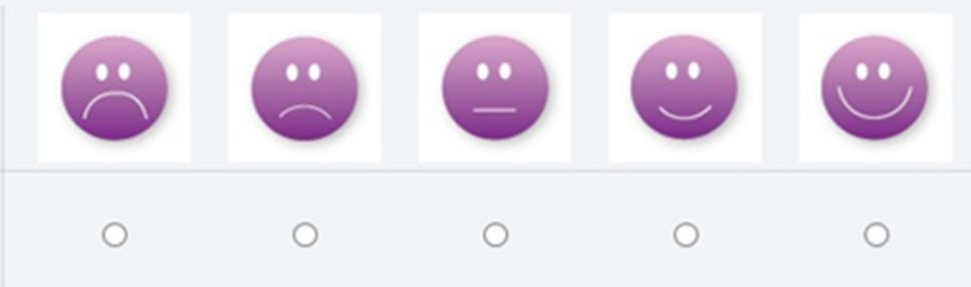
Example of the smiley face scale used for measuring the children’s affective touch ratings [adapted from^38,69^].

Participants were shown the same series of videos as in Experiment 1. These videos were shown using a custom script in PsychoPy^71^. Immediately after viewing each clip a new screen appeared where participants were asked to rate using a smiley face scale (previously validated for use in measuring affective touch ratings with young children by^38,69^, see Fig. 5]: (1) How nice do you think that was for the person being touched? (2) How much would you like to be touched like that? The two questions always appeared in the same order, each on a new screen, with question 2 appearing directly after the response to question 1 was made. They were designed to probe expectations of how touch is perceived by others versus self and worded in a child friendly lexicon in comparison to Experiment 1.

To ensure the children could use the scale effectively, before viewing and rating the videos, they completed a series of six practice trials. Here they were shown a randomised series of pictures each depicting a food that children typically find pleasant (sweets, French fries and chocolate) or unpleasant (mushrooms, Brussels sprouts and tomatoes). Children were asked to use the smiley face scale to rate how much they personally liked or did not like each food. Additionally, on half of the practice trials, children were also asked to rate how much a member of their family liked that food. This ensured that they understood both how to use the scale and that others might have different preferences to them.

### Procedure

The test procedure differed slightly for the two groups. For the autistic group, testing took place in psychology laboratories at Liverpool John Moores University. A parent was present in the test room throughout the session. During the testing, parents were asked to complete the Sensory Profile questionnaire. Participants in the control group completed the experiment at school on a one-to-one basis with the experimenter, away from their classroom.

First, participants completed the BPVS, and then they received training on the use of the rating scale (Figure 5). Participants then began the ratings task with the practice (food) trials, at this stage they were asked to say why they were choosing that particular face on the scale so the researcher could determine that the child understood the scale. Participants then watched the touch videos in PsychoPy^71^ presented in a random order. Immediately after viewing each one they rated how pleasant they perceived the touch to be for the person receiving it and how much they would like to be touched like that.

### Data analysis

Data were analysed using SPSS version 22. Responses to Question 1 and 2 (all *rs* > *0.4* & all *ps* < *0.0*1) were highly correlated so were averaged together for analysis. Inspection of model residuals indicated data were normally distributed. Assumptions of sphericity were not violated. To correct for multiple comparisons, Bonferroni correction was applied. Since here it is assumed, participants are rating pleasantness as a continuous variable and our data met the assumptions for parametric analyses^57,58^, ratings were analyzed using a 5 × 3 (Location × Velocity) Repeated Measures ANOVA to determine whether children’s ratings overall show the same relationship between Location and Velocity as adults’ do. Subsequently, analyses were then conducted using a 3(velocity) by 2(Group) ANOVA collapsed across Location. Diagnosis was included as a between subjects factor. Figures were drawn using R packages tidyverse^59^ and ggsignif^61^.

## Results

### Location × Velocity ANOVA

**Figure 6:**
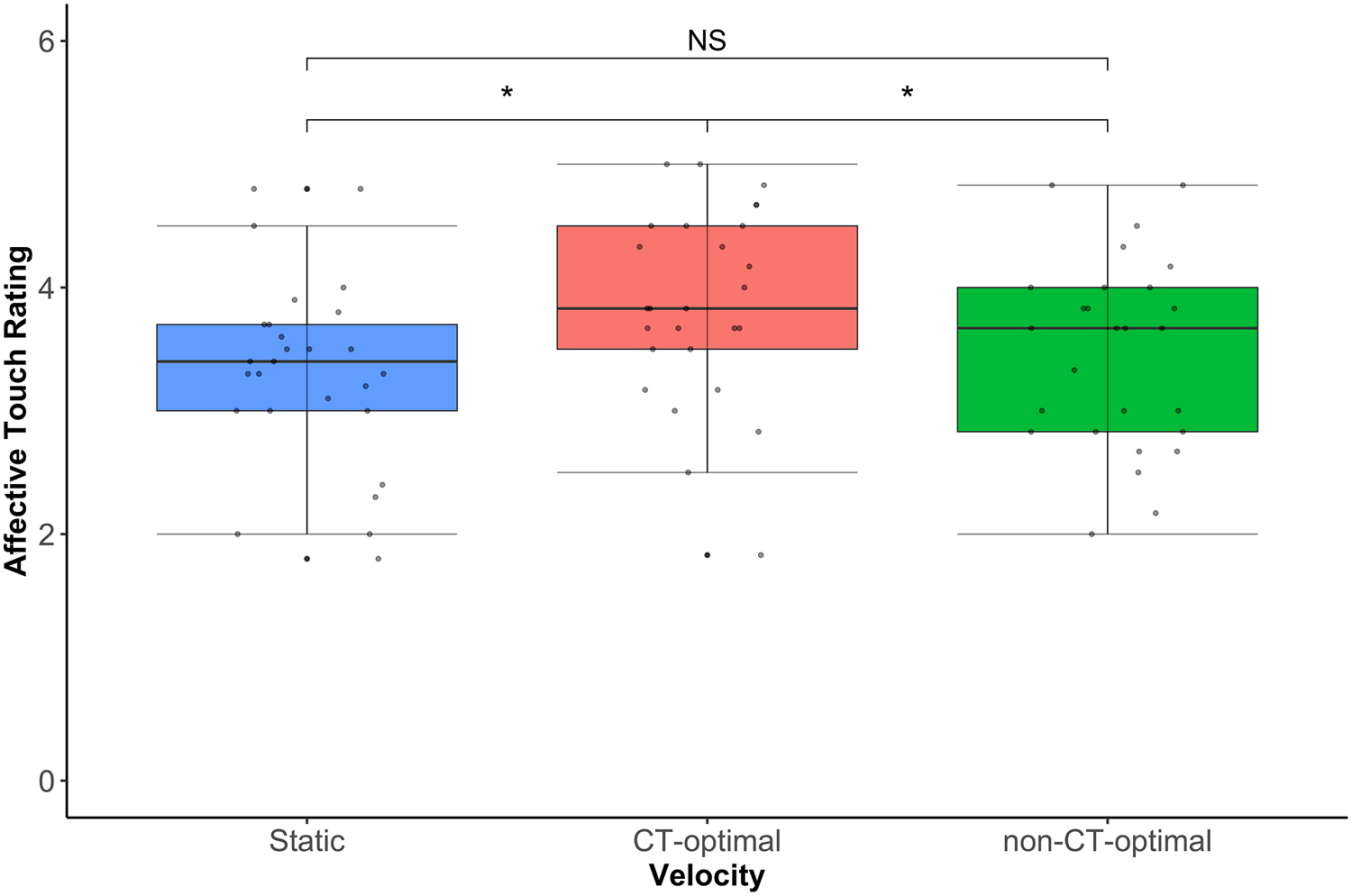
Mean affective touch ratings made by children over 3 velocities. There was a main effect of velocity and analysis of simple main effects confirmed ratings of slow CT-optimal (~ 3 cm/s) touch were significantly higher than ratings of either static or Non-CT optimal (~ 30 cm/s) touch (bars ± 95% confidence).

A repeated measures ANOVA with the factors Location (x5) × Velocity (x3) revealed a significant main effect of Velocity F(2,52)= 3.57, *p*<*.*05*,* η_p_2=.12, reflecting significantly higher affective ratings of touch at slow CT-optimal (~3cm/s), than either static (*p*<*.*01) or fast (~30cm/s) (*p*<*.*01) touch. However, there was no significant main effect of Location F(4,104)= 2.12, *p*>*.*05 *,* η_p_2=.08 nor a Location by Velocity interaction F(8,208)= .53, *p*>*.*05*,* η_p_2=.02 (Figure 6)*.*

### Group (autism/TD) × Velocity (Static/CT optimal/nonCT optimal)

**Figure 7:**
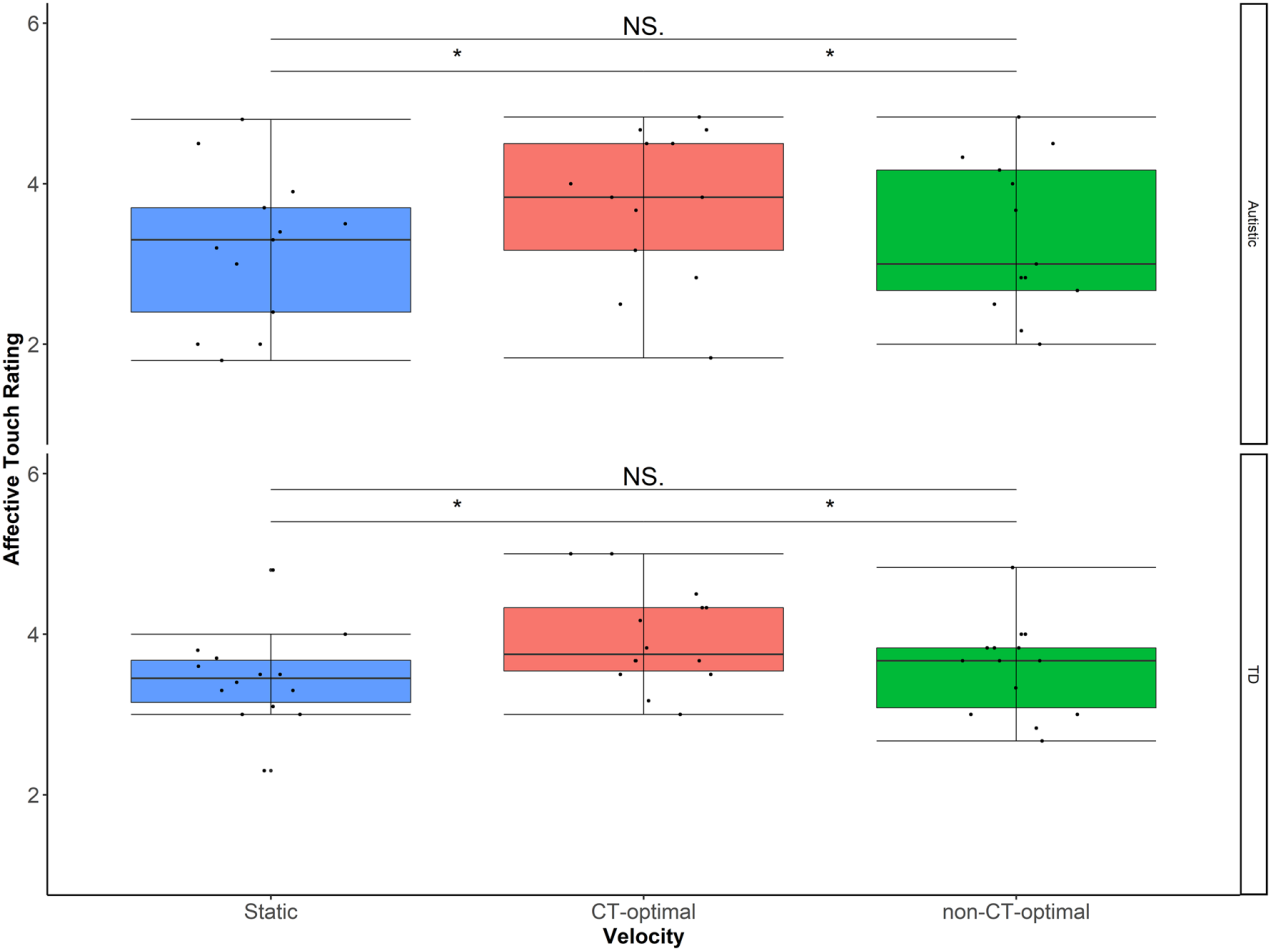
Data were collapsed across location and a group × velocity analysis was completed to show the main effect of Velocity. Here videos showing touch at CT-optimal velocities were rated higher than those depicting Static or non-CT-optimal velocity stroking and no differences between autistic children or typically developing children (bars ± 95% confidence).

As there was a main effect of Velocity but not Location in the full analysis here a 2 Group (autism/control Group) × 3 Velocity (Static/CT optimal/nonCT optimal) model was run on the data. Here there was a significant main effect of Velocity F(2,50)= 11.58, *p*<*.*001*,* η_p_2=.32 but no main effect of or Group F(1,25)= .73, *p*>*.*05*,* η_p_2=.03. Simple main effects analysis revealed that CT-optimal touch (M=3.85, SD=.78) is rated higher than both Static (M=3.32, SD=.77, *p*<*.*01) and non-CT-optimal (M=3.47, SD=.77, *p*<*.*05) Velocities (Figure 7).

For the autism group, parental report data from the Sensory Profile (Dunn et al., 1999) were correlated with touch ratings for each Velocity to determine whether sensitivity to touch affected participant’s affective touch ratings. Here there were no significant correlations between touch processing score and affective touch ratings for touch at either Static (r=.34, *p*=*.*25) CT-optimal velocity (r=.28, *p*=*.*36) or a non-CT-optimal velocity (r=.36, *p*=*.*23) touch. These data show that parent reported touch processing scores have no relationship to the child’s rating of touch preference.

## Discussion.

Consistent with studies of directly felt touch^21^, here young children’s ratings of vicariously experienced social touch showed the previously reported relationship between velocity and perceived pleasantness, with CT-optimal touch being rated higher than non-CT optimal touch^50,51^. However, in contrast to adults, the children’s ratings did not differ according to touch location. That is, they did not rate touch on the back higher than any other location and their ratings did not differ between glabrous and hairy skin sites. Though this could reflect limited power to detect effects with the relatively small sample size in the present experiment, the findings are consistent with another larger study (N = 44) we conducted with similar aged children using these stimuli^55^, where children’s ratings also varied by velocity, but not location. These results suggest that expectations about the pleasantness of social touch develop with experience.

Counter to our hypothesis, children with a diagnosis of autism did not show blunted ratings of CT optimal touch in comparison to their typically developing peers. Indeed, there was no effect of diagnosis on any aspect of touch ratings. This is despite the fact parental reports of touch processing on the sensory profile indicated these children showed differences in somatosensory processing (M = 54.23, SD = 12.6) compared to registered norms (i.e., scores less than 64 are considered different from TD individuals). This lack of relationship between vicarious ratings and parental reports of tactile sensitivity is consistent with a previous psychophysical study where high functioning children with a diagnosis of autism’s affective ratings of tactile stimuli did not correlate significantly with parental reports of sensory symptoms^38^. The authors speculate this lack of relationship could reflect differences between the controlled predictability of experimental stimuli versus the unpredictability of tactile experiences in daily life.

The lack of location specific effects in the present study are inconsistent with previous reports that experimenter-delivered affective (pleasant and unpleasant) touch to autistic children elicits more severe defensiveness when delivered on hairy (face and arm) rather than on glabrous skin sites (palm)^38^. Here, the self-report ratings were correlated with experimenter coding of tactile defensiveness. Thus, it seems unlikely our null result reflects the children’s lack of ability to use the scale.

A potential limitation of the present study is that the videos depict touch between two adults. Therefore, future studies should consider using stimuli showing more relevant tactile context such as parent/caregiver delivered touch (adult-to-child) or peer-to-peer touch between child actors. However, previous work on vicarious responses to pain have found that children aged 7–11 are able to accurately rate animated clips showing adults experiencing pain inflicted by another adult actor^72^. These stimuli, like ours, lacked broader social cues such as faces. Importantly, when viewing the stimuli, the children, like adults, showed increased haemodynamic responses in neural circuits involved in processing the first-hand experience of pain, as well as areas involved in processing social behaviour. This previous work suggests, despite age dependent changes in activity of brain regions involved in empathy^73^, children in our sample had the developmental capacity to decode and affectively rate social tactile interactions such as those depicted. A further limitation was that for the children in the typically developing group we did not collect sensory profile information. The aim of this measure was to determine whether the autistic group had high levels of sensory sensitivities, but it would be beneficial to see how these results relate to the typically developing group.

## General discussion and conclusion

The results of these two studies replicate previous work showing that adult’s vicarious ratings of social touch vary according to both velocity and location^19,51^, with CT optimal velocity touch being rated higher (thus more positive/desired) than static or faster, non-CT optimal velocity touch at all skin sites studied, except the palm. In contrast, while children were able to differentiate between velocities of touch, rating CT optimal touch as more pleasant than non-CT optimal touch, their ratings did not differ by touch location. That is, unlike adults, they didn’t rate touch on the back as more pleasant than any other skin site and their ratings of touch on the palm showed the same inverted U-shaped relationship between velocity and pleasantness as all hairy skin sites tested. These data suggest that, in contrast to our hypotheses, a diagnosis of autism did not appear to affect ratings in comparison to typically developing children. Comparatively, supporting the hypothesis, individuals with high levels of autistic traits showed less preference for touch in general and specifically showed altered preference for touch on the palm compared to individuals with low levels of autistic traits.

These findings contrast with a previous psychophysical study which reported a negative relationship between autistic traits and sensitivity to the rewarding value of CT targeted touch^21^. In the literature overall, the relationship between autistic traits or diagnosis of autism and affective responses to touch are more consistently seen in children^38^ than adults and in neural^33,36^ than psychophysical studies^35,37^. It can be concluded that here awareness of touch preferences in others is present in autistic children as well as typically developing children. They therefore show the same pattern of ratings for videos of touch regardless of diagnosis; however, children appear to consider the whole body rather than individual body sites when considering the pleasantness of touch. Our findings demonstrate the need for a wider range of sensory domains to be considered when examining the nature of empathy in autistic children and adults.

The perception of touch can be modulated by a range of top-down factors, with past tactile interactions shaping future touch^74^. Our findings indicate adults can discriminate varying affective qualities of social tactile interactions more sensitively than children. This difference could be due either to the children’s social-cognitive limitations or their more limited social history. Previous studies in adults have shown that vicarious ratings of affective touch are shaped by the personal experiences of the viewer^49,50^. Thus, the fact there were no differences between the autism group and neurotypical controls in hedonic ratings, despite parental reports of somatosensory sensitivities in autism group, suggests young children may not engage the necessary the cognitive processes to accurately use their own experiences to evaluate and inform their expectations about the experiences of others^75^. Though it may also perhaps reflect the limited variability of the scale we used, decreasing power to detect subtle differences between groups^38^.

In addition to the overall effects of autism presentation on ratings of vicarious pleasantness of touch, there were also noteworthy effects regarding the location of touch on ratings. In adults, individuals with high levels of autistic traits rated vicariously experienced touch as less pleasant than a group with low levels of self-reported traits. These differences were not velocity but location specific and driven by the high AQ group’s lower ratings of touch on glabrous skin (palm) where typically these ratings are comparable and driven by velocity^76^. It is therefore possible these findings are underpinned by group differences in experiences of discriminative rather than affective aspects of touch. The palm is more densely innervated by myelinated Aβ low threshold mechanoreceptors (LTMRs) than the other skin sites tested. Though the contribution of Aβ afferents to the emotional processing of touch have not been widely explored [though see^77^], it could be that perceptions of touch intensity on the palm are greater amongst adults with high levels of autistic traits and this drives the blunted affective ratings reported. The implications of this might include a reduced drive to engage with either intimate interpersonal touch such as hand holding or typical business interactions, such as shaking hands. It is important to note that brain processing of pleasant touch differs between hairy and glabrous skin^77^. Contrasting slow brush stroking on the forearm with slow brush stroking on the palm, revealed significant activations of the posterior insular cortex and mid-anterior orbitofrontal cortex, while the opposite contrast showed a significant activation of the somatosensory cortices. Therefore, the location specific findings in the present study may reflect differences in processing sensory rather than affective aspects of the stimulus.

Though both myelinated and unmyelinated C-type LTMRs respond to static touch^15,78^, psychophysical studies of affective touch typically only consider dynamic stimuli, which reliably, on both the hairy arm and glabrous palmar skin, elicit an inverted U-shaped relationship between stimulus velocity and perceived pleasantness^14,18^. Thus, it isn’t clear how the vicarious rating of static touch on the palm we report here relate to ratings of directly experienced stimuli on the same skin site. This is important because contextually, palmar touch is a key aspect of human tactile interactions, it is the primary skin site used in active social touch, such as holding hands or providing a reassuring pat on the back^79–82^. Psychophysical data would help differentiate bottom up sensory from top-down cognitive effects on our ratings. This would be insightful because there are individual differences in attitudes and experiences towards social touch, based in part on social history^50,83,84^, and the acceptability of social touch depends on the nature of the bonds between toucher and receiver^85^. Given the AQ has been conceptualised to be primarily a measure of trait sociability^86^, the differences in glabrous skin ratings in our high and low AQ group may reflect differences in trait sociability rather than sensory perception, with the more positive attitudes and experiences of social touch in the low AQ group driving higher hedonic ratings of touch in this location. Future work could provide more detailed social context to the videos to determine whether establishing the relationship between the actors in the scenario depicted has any effect on the ratings of participants with high scores on the AQ, or indeed how adults with a diagnosis of autism rate these stimuli.

A further limitation of the experiments reported here is that participant’s trait empathic ability was not measured. The empathic ability of participants has been shown to affect their performance in tasks of embodiment or vicarious experience^87–89^ therefore, it would have been prudent to consider how this may have affected variation in participants’ ratings of the videos. In individuals with autism, while cognitive empathy, the ability to understand and respond appropriately to others’ mental states and emotions, may be different, affective empathy, the ability to respond with an appropriate emotion to others’ mental states or feelings, appears typical^8,90,91^. Here, though our stimuli involved two actors, participants were asked to consider the emotional responses of the person being touched, which we anticipate can be decoded simply through affective resonance with the individual depicted, a capacity that is functional early in life, and does not require higher order representation of the toucher’s intentions^92^. However, since previous studies have shown that trait empathy predicts both neural responses to, and affective ratings of, social touch it would be insightful to determine its relationship with vicarious ratings of our stimuli.

## Conclusion

The studies reported here show vicarious ratings of social-affective touch develop with age but vary little, at least in autistic individuals without a co-occurring learning disability, in terms of autism diagnosis or self-reported autistic traits. Future work is needed to determine when, developmentally, location specific preferences emerge and how these relate to ratings of directly felt touch.

## References

[1] American Psychiatric Association. *Diagnostic and statistical manual of mental disorders : DSM-5.* Washington, D.C: American Psychiatric Association (2013).

[2] Dawson G, Watling R (2000). Interventions to facilitate auditory, visual, and motor integration in autism: a review of the evidence. J. Autism Dev. Disord..

[3] Tomchek SD, Dunn W (2007). Sensory processing in children with and without autism: a comparative study using the short sensory profile. Am. J. Occup. Ther..

[4] Foss-Feig JH, Heacock JL, Cascio CJ (2012). Tactile responsiveness patterns and their association with core features in autism spectrum disorders. Res. Autism Spectrum Disord..

[5] Mikkelsen M, Wodka EL, Mostofsky SH, Puts NAJ (2018). Autism spectrum disorder in the scope of tactile processing. Dev. Cognit. Neurosci..

[6] Thye MD, Bednarz HM, Herringshaw AJ, Sartin EB, Kana RK (2017). The impact of atypical sensory processing on social impairments in autism spectrum disorder. Dev. Cognit. Neurosci..

[7] Haigh SM (2018). Variable sensory perception in autism. Eur. J. Neurosci..

[8] Robertson CE, Baron-Cohen S (2017). Sensory perception in autism. Nat. Rev. Neurosci..

[9] Cascio CJ (2010). Somatosensory processing in neurodevelopmental disorders. J. Neurodev. Disord..

[10] McGlone F, Wessberg J, Olausson H (2014). Discriminative and affective touch: sensing and feeling. Neuron.

[11] McGlone F, Reilly D (2010). The cutaneous sensory system. Neurosci. Biobehav. Rev..

[12] Nagi, S. S., Marshall, A. G., Makdani, A., Jarocka, E., Liljencrantz, J., Ridderström, M. & Olausson, H. *An ultrafast system for signaling mechanical pain in human skin*. *Sci. Adv* (Vol. 5) (2019). Retrieved from http://advances.sciencemag.org/. 10.1126/sciadv.aaw1297 PMC6609212 31281886

[13] Bishop GH, Landau WM (1958). Evidence for a double peripheral pathway for pain. Science.

[14] Löken LS, Wessberg J, Morrison I, McGlone F, Olausson H (2009). Coding of pleasant touch by unmyelinated afferents in humans. Nat. Neurosci..

[15] Vallbo ÅB, Olausson H, Wessberg J, Ackerley R, Wasling HB, Liljencrantz J, Thonnard J (1999). Unmyelinated afferents constitute a second system coding tactile stimuli of the human hairy skin unmyelinated afferents constitute a second system coding tactile stimuli of the human hairy skin. J. Neurophysiol..

[16] Björnsdotter M, Löken L, Olausson H, Vallbo A, Wessberg J (2009). Somatotopic organization of gentle touch processing in the posterior insular cortex. J. Neurosci.: Off. J. Soc. Neurosci..

[17] Craig AD (2003). Interoception: the sense of the physiological condition of the body. Curr. Opin. Neurobiol..

[18] Essick GK, James A, McGlone FP (1999). Psychophysical assessment of the affective components of non-painful touch. NeuroReport.

[19] Morrison I, Löken LS, Minde J, Wessberg J, Perini I, Nennesmo I, Olausson H (2011). Reduced C-afferent fibre density affects perceived pleasantness and empathy for touch. Brain: A J. Neurol..

[20] Olausson H, Wessberg J, Morrison I, McGlone F, Vallbo A (2010). The neurophysiology of unmyelinated tactile afferents. Neurosci. Biobehav. Rev..

[21] Croy I, Luong A, Triscoli C, Hofmann E, Olausson H, Sailer U (2016). Interpersonal stroking touch is targeted to C tactile afferent activation. Behav. Brain Res..

[22] Van Puyvelde M, Collette L, Gorissen AS, Pattyn N, McGlone F (2019). Infants autonomic cardio-respiratory responses to nurturing stroking touch delivered by the mother or the father. Front. Physiol..

[23] Van Puyvelde M, Gorissen AS, Pattyn N, McGlone F (2019). Does touch matter? The impact of stroking versus non-stroking maternal touch on cardio-respiratory processes in mothers and infants. Physiol. Behav..

[24] Adolphs R (2003). Cognitive neuroscience: cognitive neuroscience of human social behaviour. Nat. Rev. Neurosci..

[25] Adolphs R (2008). The Social brain: neural basis of social knowledge. Annu. Rev. Psychol..

[26] Blakemore S-J (2008). The social brain in adolescence. Nat. Rev. Neurosci..

[27] Gallagher HL, Frith CD (2003). Functional imaging of ‘theory of mind’. Trends Cogn. Sci..

[28] Gordon I, Vander Wyk BC, Bennett RH, Cordeaux C, Lucas MV, Eilbott J, Pelphrey K (2013). Oxytocin enhances brain function in children with autism. Proc. Natl. Acad. Sci. USA.

[29] Brauer J, Xiao Y, Poulain T, Friederici AD, Schirmer A (2016). Frequency of maternal touch predicts resting activity and connectivity of the developing social. Brain.

[30] Björnsdotter M, Davidovic M, Karjalainen L, Starck G, Olausson H, Wentz E (2018). Grey matter correlates of autistic traits in women with anorexia nervosa. J. Psychiatry Neurosci..

[31] Crucianelli L, Cardi V, Treasure J, Jenkinson PM, Fotopoulou A (2016). The perception of affective touch in Anorexia Nervosa. Psychiatry Res..

[32] Kaiser MD, Yang DY-J, Voos AC, Bennett RH, Gordon I, Pretzsch C, Pelphrey KA (2015). Brain mechanisms for processing affective (and nonaffective) touch are atypical in autism. Cereb. Cortex.

[33] Voos AC, Pelphrey KA, Kaiser MD (2013). Autistic traits are associated with diminished neural response to affective touch. Soc. Cognit. Affect. Neurosci..

[34] Cascio CJ, Moore D, McGlone F (2018). Social touch and human development. Dev. Cogn. Neurosci..

[35] Perini I, Gustafsson PA, Igelström K, Jasiunaite-Jokubaviciene B, Kämpe R, Mayo LM, Molander J, Olausson H, Zetterqvist M, Heilig M (2021). Altered relationship between subjective perception and central representation of touch hedonics in adolescents with autism spectrum disorder. Transl. Psychiatry.

[36] Haggarty CJ, Malinowski P, McGlone FP, Walker SC (2020). Autistic traits modulate cortical responses to affective but not discriminative touch. Eur. J. Neurosci..

[37] Cascio C, McGlone F, Folger S, Tannan V, Baranek G, Pelphrey KA, Essick G (2008). Tactile perception in adults with autism: a multidimensional psychophysical study. J. Autism Dev. Disord..

[38] Cascio CJ, Lorenzi J, Baranek GT (2016). Self-reported pleasantness ratings and examiner-coded defensiveness in response to touch in children with ASD: effects of stimulus material and bodily location. J. Autism Dev. Disord..

[39] Decety J, Jackson PL (2004). The functional architecture of human empathy. Behav. Cognit. Neurosci. Rev..

[40] Jackson PL, Rainville P, Decety J (2006). To what extent do we share the pain of others? Insight from the neural bases of pain empathy. Pain.

[41] Morrison I, Tipper SP, Fenton-Adams WL, Bach P (2013). “Feeling” others’ painful actions: the sensorimotor integration of pain and action information. Hum. Brain Mapp..

[42] Singer T, Seymour B, O’Doherty J, Dolan RJ, Kaube H, Frith CD, Frith CD (2004). Empathy for pain involves the affective but not sensory components of pain. Science (New York, NY).

[43] Lamm C, Porges EC, Cacioppo JT, Decety J (2008). Perspective taking is associated with specific facial responses during empathy for pain. Brain Res..

[44] Bird G, Silani G, Brindley R, White S, Frith U, Singer T (2010). Empathic brain responses in insula are modulated by levels of alexithymia but not autism. Brain.

[45] Fan YT, Chen C, Chen SC, Decety J, Cheng Y (2014). Empathic arousal and social understanding in individuals with autism: Evidence from fMRI and ERP measurements. Soc. Cognit. Affect. Neurosci..

[46] Hadjikhani N, Zürcher NR, Rogier O, Hippolyte L, Lemonnier E, Ruest T, Prkachin KM (2014). Emotional contagion for pain is intact in autism spectrum disorders. Transl. Psychiatry.

[47] Milton DE (2012). On the ontological status of autism: the ‘double empathy problem’. Disabil. Soc..

[48] Bolognini N, Rossetti A, Fusaro M, Vallar G, Miniussi C (2014). Sharing social touch in the primary somatosensory cortex. Curr. Biol..

[49] Keysers C, Kaas JH, Gazzola V (2010). Somatosensation in social perception. Nat. Rev. Neurosci..

[50] Morrison I, Björnsdotter M, Olausson H (2011). Vicarious responses to social touch in posterior insular cortex are tuned to pleasant caressing speeds. J. Neurosci.: Off. J. Soc. Neurosci..

[51] Devine SL, Walker SC, Makdani A, Stockton ER, McFarquhar MJ, McGlone FP, Trotter PD (2020). Childhood adversity and affective touch perception: a comparison of United Kingdom care leavers and non-care leavers. Front. Psychol..

[52] Walker SC, Trotter PD, Woods A, McGlone F (2017). Vicarious ratings of social touch reflect the anatomical distribution & velocity tuning of C-tactile afferents: a hedonic homunculus?. Behav. Brain Res..

[53] Jönsson EH, Kotilahti K, Heiskala J, Wasling HB, Olausson H, Croy I, Nissilä I (2018). Affective and non-affective touch evoke differential brain responses in 2-month-old infants. Neuroimage.

[54] Tuulari JJ, Scheinin NM, Lehtola S, Merisaari H, Saunavaara J, Parkkola R, Björnsdotter M (2019). Neural correlates of gentle skin stroking in early infancy. Dev. Cognit. Neurosci..

[55] Croy I, Sehlstedt I, Wasling HB, Ackerley R, Olausson H (2019). Gentle touch perception: from early childhood to adolescence. Devel. Cognit. Neurosci..

[56] Haggarty, C., Trotter, P., McGlone, F. & Walker, S. Children’s vicarious ratings of social touch are tuned to the velocity but not the location of a caress (2021). PsyArXiv, 10.31234/osf.io/396hb 10.1371/journal.pone.0256303 PMC8389448 34437583

[57] Baron-cohen S, Wheelwright S, Skinner R, Martin J, Clubley E (2001). The autism-spectrum quotient (AQ ): evidence from Asperger Syndrome/high-functioning autism, males and females, scientists and mathematicians. J. Autism Dev. Disord..

[58] Mircioiu C, Atkinson J (2017). A comparison of parametric and non-parametric methods applied to a likert scale. Pharmacy.

[59] Woodbury-Smith MR, Robinson J, Wheelwright S, Baron-Cohen S (2005). Screening adults for asperger syndrome using the AQ: a preliminary study of its diagnostic validity in clinical practice. J. Autism Dev. Disord..

[60] Velleman PF, Wilkinson L (1993). Nominal, ordinal, interval and ratio typologies are misleading. Am. Stat..

[61] Wickham, H. tidyverse: Easily Install and Load the 'Tidyverse'. R package version 1.2.1. (2017). https://CRAN.R-project.org/package=tidyverse.

[62] Constantin A, Patil I (2021). ggsignif: R package for displaying significance brackets for 'ggplot2'. PsyArxiv.

[63] Kennedy WR, Wendelschafer-Crabb G, Polydefkis M, McArthur JC (2005). Pathology and quantitation of cutaneous innervation. Peripheral Neuropathy.

[64] Liu Q, Vrontou S, Rice FL, Zylka MJ, Dong X, Anderson DJ (2007). Molecular genetic visualization of a rare subset of unmyelinated sensory neurons that may detect gentle touch. Nat. Neurosci..

[65] Cascio CJ, Foss-feig JH, Heacock JL, Newsom CR, Cowan RL, Benningfield MM, Cao A (2012). Response of neural reward regions to food cues in autism spectrum disorders. J. Neurodev. Disord..

[66] Fairhurst MT, Löken L, Grossmann T (2014). Physiological and behavioral responses reveal 9-month-old infants’ sensitivity to pleasant touch. Psychol. Sci..

[67] Kida T, Shinohara K (2013). Gentle touch activates the anterior prefrontal cortex: an NIRS study. Neurosci. Res..

[68] Björnsdotter M, Gordon I, Pelphrey KA, Olausson H, Kaiser MD (2014). Development of brain mechanisms for processing affective touch. Front. Behav. Neurosci..

[69] Croy I, Sehlstedt I, Wasling HB, Ackerley R, Olausson H (2017). Gentle touch perception: from early childhood to adolescence. Dev. Cogn. Neurosci..

[70] American Psychiatric Association. *Diagnostic and Statistical Manual of Mental Disorders* (4th ed., T). Washington DC: Author (2000). 10.1176/appi.books.9780890423349

[71] Dunn LM, Dunn LM, Whetton K, Burley J (1997). British Picture Vocabulary Scale.

[72] Pierce JW (2007). PsychoPy—psychophyics software in Python. J. Neurosci. Methods.

[73] Decety J, Michalska KJ, Akitsuki Y (2008). Who caused the pain? An fMRI investigation of empathy and intentionality in children. Neuropsychologia.

[74] Michalska KJ, Kinzler KD, Decety J (2013). Age-related sex differences in explicit measures of empathy do not predict brain responses across childhood and adolescence. Dev. Cogn. Neurosci..

[75] Ellingsen DM, Leknes S, Løseth G, Wessberg J, Olausson H (2016). The neurobiology shaping affective touch: Expectation, motivation, and meaning in the multisensory context. Front. Psychol..

[76] Lombardo MV, Baron-Cohen S (2011). The role of the self in mindblindness in autism. Conscious. Cogn..

[77] Eriksson Hagberg E, Ackerley R, Lundqvist D, Schneiderman J, Jousmäki V, Wessberg J (2019). Spatio-temporal profile of brain activity during gentle touch investigated with magnetoencephalography. Neuroimage.

[78] McGlone F, Olausson H, Boyle J, Jones-Gotman M, Dancer C, Guest S, Essick G (2012). Touching and feeling: differences in pleasant touch processing between glabrous and hairy skin in humans. Eur. J. Neurosci..

[79] Abraira V, Ginty D (2013). The sensory neurons of touch. Neuron.

[80] Coan JA, Schaefer HS, Davidson RJ (2006). Lending a hand of the neural response to threat. Psychol. Sci..

[81] Fisher JD, Rytting M, Heslin R (1976). Hands touching hands: affective and evaluative effects of an interpersonal touch. Access. Sociom..

[82] Johnson SM, Moser MB, Beckes L, Smith A, Dalgleish T, Halchuk R, Coan JA (2013). Soothing the threatened brain: leveraging contact comfort with emotionally focused therapy. PLoS ONE.

[83] Weekes DP, Kagan SH, James K, Seboni N (1993). The phenomenon of hand holding as a coping strategy in adolescents experiencing treatment-related pain. J. Pediatr. Oncol. Nurs..

[84] Sailer U, Ackerley R (2017). Exposure shapes the perception of affective touch. Dev. Cognit. Neurosci..

[85] Trotter PD, Mcglone F, Reniers RLEP, Deakin JFW (2018). Construction and validation of the touch experiences and attitudes questionnaire (TEAQ): a self-report measure to determine attitudes toward and experiences of positive touch. J. Nonverbal Behav..

[86] Suvilehto JT, Glerean E, Dunbar RIM, Hari R, Nummenmaa L (2015). Topography of social touching depends on emotional bonds between humans. Proc. Natl. Acad. Sci..

[87] Hoekstra RA, Bartels M, Cath DC, Boomsma DI (2008). Factor structure, reliability and criterion validity of the autism-spectrum quotient (AQ): a study in Dutch population and patient groups. J. Autism Dev. Disord..

[88] Kaplan JT, Iacoboni M (2006). Getting a grip on other minds: mirror neurons, intention understanding, and cognitive empathy. Soc. Neurosci..

[89] Minio-Paluello I, Baron-Cohen S, Avenanti A, Walsh V, Aglioti SM (2009). Absence of embodied empathy during pain observation in asperger syndrome. Biol. Psychiat..

[90] Rueda P, Fernández-Berrocal P, Baron-Cohen S (2014). Dissociation between cognitive and affective empathy in youth with Asperger Syndrome. Eur. J. Dev. Psychol..

[91] Dziobek I, Rogers K, Fleck S, Bahnemann M, Heekeren HR, Wolf OT, Convit A (2008). Dissociation of cognitive and emotional empathy in adults with Asperger syndrome using the multifaceted empathy test (MET). J. Autism Dev. Disord..

[92] Mazza M, Pino MC, Mariano M, Tempesta D, Ferrara M, De Berardis D, Valenti M (2014). Affective and cognitive empathy in adolescents with autism spectrum disorder. Front. Hum. Neurosci..

